# Similar response profile to neoadjuvant chemotherapy, but different survival, in inflammatory *versus* locally advanced breast cancers

**DOI:** 10.18632/oncotarget.19732

**Published:** 2017-07-31

**Authors:** Audrey Monneur, Anthony Goncalves, Marine Gilabert, Pascal Finetti, Carole Tarpin, Christophe Zemmour, Jean-Marc Extra, Agnès Tallet, Eric Lambaudie, Jocelyne Jacquemier, Gilles Houvenaeghel, Jean-Marie Boher, Patrice Viens, François Bertucci

**Affiliations:** ^1^ Département d’Oncologie Médicale, Centre de Recherche en Cancérologie de Marseille (CRCM), Institut Paoli-Calmettes, Marseille, France; ^2^ Faculté de Médecine, Aix-Marseille Université, Marseille, France; ^3^ Département de la Recherche Clinique et de l’Innovation, Unité de Biostatistiques et Méthodologie, INSERM, IRD, Institut Paoli-Calmettes, Aix-Marseille Université, Marseille, France; ^4^ Département de Radiothérapie, Institut Paoli-Calmettes, Marseille, France; ^5^ Département d’Oncologie Chirurgicale, Institut Paoli-Calmettes, Marseille, France; ^6^ Département d’Anatomopathologie, Institut Paoli-Calmettes, Marseille, France

**Keywords:** breast cancer, chemotherapy, inflammatory breast cancer, pathological response, survival

## Abstract

Inflammatory breast cancer (IBC) is a very aggressive form of breast cancer, as compared to locally advanced breast cancer (LABC). Neoadjuvant chemotherapy followed by surgery is the standard treatment in both cases. Whether IBC is less chemosensitive than LABC remains unclear. We retrospectively compared the rate of pathological complete response (pCR) to neoadjuvant chemotherapy in IBC and LABC. Methods: Patients with IBC or LABC treated with neoadjuvant anthracycline-based chemotherapy followed by surgery were selected from our institutional database. The primary endpoint was the pCR rate, defined as absence of invasive tumor in breast and axillary lymph nodes. Results: A total of 450 patients were included, 144 with IBC and 306 with LABC. The pCR rate was similar between the two groups, in the whole population (31%) and in each molecular subtype separately. Univariate analyses for pCR in IBC and LABC separately identified the same predictive variables, except the pathological type that was associated with pCR in LABC only, but not in IBC. IBC patients displayed shorter 5-year metastasis-free survival and overall survival than LABC patients in the whole population (57% and 69% *versus74%* and 88% respectively), and in each molecular subtype separately. The IBC phenotype was an independent prognostic feature. Similarly, IBC patients displayed shorter 5-year loco-regional relapse-free survival than LABC patients (86% *versus* 95%). Conclusions: Similar pCR rates to chemotherapy were found in IBC and LABC, suggesting that IBC is not less chemosensitive than LABC. Survival was shorter in IBC, suggesting that the corresponding poorer prognosis is more due to a higher metastatic risk and/or other feature(s) than to a lesser chemosensitivity.

## INTRODUCTION

Inflammatory breast cancer (IBC) is the most aggressive form of breast cancer. IBC is classified as cT4d according to the AJCC TNM staging system [1]. Before the last three decades, patients with IBC were treated with surgery and/or radiotherapy and the prognosis was disastrous with an overall survival inferior to 5%, due to nearly constant metastatic relapse [2]. The survival has been greatly improved by the introduction of neoadjuvant chemotherapy with successive additions of anthracyclines, then taxanes for all patients, then trastuzumab and more recently pertuzumab for HER2+ disease. However despite this multimodal therapy, the 5-year survival still remains close to 50-60% [1]. Such poor prognosis is of course due in a large part to the strong metastatic potential of disease [3]. But IBC is also classically considered less sensitive to chemotherapy [4], hormone therapy [5] and radiotherapy [6] than non-IBC. In fact, whether IBC is less sensitive to chemotherapy than non-IBC remains unclear.

The model of neoadjuvant chemotherapy provides opportunity for assessing the tumor chemosensitivity in non-metastatic patients [7, 8]. Patients are treated generally with six or eight cycles of anthracycline-based regimen before surgery [9]. Pathological analysis of the operative specimen provides the degree of pathological response, defined as complete (pCR) or not complete, and in general, the achievement of pCR is a favorable prognostic factor [10]. Neoadjuvant chemotherapy is the treatment of choice in patients with IBC, but also for patients with locally advanced breast cancer (LABC), and has become a standard option for primary operable breast cancer (OBC) in patients candidate for adjuvant chemotherapy [11]. Current regimens do not differ significantly between IBC, LABC and OBC [12].

Because IBC is clinically and biologically different from operable or locally advanced non-IBC, patients with IBC have often been excluded from neoadjuvant clinical trials dedicated to non-IBC. Furthermore, few prospective trials have been dedicated to IBC because of the low incidence of disease. Thus, prospective data on IBC are rare in the literature, as well as the direct comparison between IBC *versus* non-IBC with respect to the response to chemotherapy. Here, we retrospectively compared the pCR rate to neoadjuvant chemotherapy in a large series of patients with IBC and LABC treated in our institution.

## RESULTS

### Patients’ baseline characteristics and treatment

A total of 450 non-metastatic breast cancer patients treated in our institution between 1992 and 2015 with anthracycline-based neoadjuvant chemotherapy were included, comprising 144 patients with IBC and 306 with LABC (Table 1). Compared with patients with LABC, patients with IBC showed less frequently positive clinical axillary lymph node status (cN1-3: 70% *vs* 82%), and had more frequently dermal emboli (46% *vs* 6%), and grade 3 tumors (62% *vs* 50%). No significant difference was observed regarding age, menopausal status, pathological type, and molecular subtypes. The molecular subtype of tumors was defined as follows: HR+ for ER+ and/or PR+ and HER2- tumors, HER2+ for HER2+ tumors, and triple negative (TN) for ER-, PR- and HER2- tumors.

**Table 1:** Patients’ characteristics at baseline

Characteristics	All N=450	LABC N=306	IBC N=144	*P*-value
Median age at diagnosis (years, range)	49 (19-85)	49 (19-78)	50.5 (27-85)	0.791
Menopause				0.835
no	238 (55%)	163 (56%)	75 (54%)	
yes	192 (45%)	129 (44%)	63 (46%)	
Clinical axillary lymph node status, cN				**6.87E-03**
0	98 (22%)	56 (18%)	42 (30%)	
1-3	347 (78%)	249 (82%)	98 (70%)	
Pathological type				0.939
ductal	372 (83%)	254 (83%)	118 (83%)	
lobular	39 (9%)	26 (8%)	13 (9%)	
other	37 (8%)	26 (8%)	11 (8%)	
Pathological grade				**4.67E-02**
1	33 (8%)	23 (8%)	10 (7%)	
2	167 (39%)	125 (43%)	42 (31%)	
3	231 (54%)	146 (50%)	85 (62%)	
Molecular subtype, IHC status				0.632
HR +	208 (46%)	145 (47%)	63 (44%)	
HER2 +	144 (32%)	98 (32%)	46 (32%)	
TN	98 (22%)	63 (21%)	35 (24%)	
Dermal emboli				**1.43E-16**
no	248 (79%)	183 (94%)	65 (54%)	
yes	67 (21%)	12 (6%)	55 (46%)	
Neoadjuvant chemotherapy				**1.05E-12**
anthracycline	94 (21%)	34 (11%)	60 (42%)	
anthracycline & taxane	356 (79%)	272 (89%)	84 (58%)	
Surgery				**1.16E-14**
mastectomy	340 (76%)	202 (66%)	138 (97%)	
lumpectomy	108 (24%)	103 (34%)	5 (3%)	
Adjuvant radiotherapy				1
no	13 (3%)	9 (3%)	4 (3%)	
yes	431 (97%)	295 (97%)	136 (97%)	
Neoadjuvant/adjuvant trastuzumab				**3.63E-03**
no	335 (74%)	215 (70%)	120 (83%)	
yes	115 (26%)	91 (30%)	24 (17%)	
Adjuvant hormone therapy				0.117
no	173 (40%)	109 (37%)	64 (45%)	
yes	261 (60%)	184 (63%)	77 (55%)	

Regarding the treatment, and compared with patients with LABC, patients with IBC had received less anthracycline and taxane-based neoadjuvant chemotherapy (58% *vs* 89%), and less neoadjuvant/adjuvant trastuzumab (17% *vs* 30%). All patients had definitive surgery, with more frequent mastectomy than lumpectomy in patients with IBC, compared with LABC patients (97% *vs* 66%). The proportion of patients treated with adjuvant radiotherapy was similar between the two groups (97%), as was the proportion of patients treated with adjuvant hormone therapy (55% in IBC *vs* 63% in LABC).

### Pathological response to neo-adjuvant chemotherapy

Analysis of the 450 operative specimens showed that 141 patients had achieved pCR (31%, 95%CI (27-36)). The pCR rate was similar between patients with IBC (31%, 95%CI (24-40)) and those with LABC (31%, 95%CI (26-37); p=1, Fisher’s exact test; Table 2). In univariate analyses for prediction of pCR, the ductal type (*vs* lobular or other), the HER2+ and TN molecular subtypes (*vs* HR+ subtype), the delivery of neoadjuvant trastuzumab (*vs* no delivery), and the delivery of anthracycline and taxane-based regimen of neoadjuvant chemotherapy (*vs* anthracycline without taxane) were associated with higher pCR rate (Table 3). The other variables (age, cN status, pathological grade, and IBC/LABC stage) were not associated with pCR. The Odds Ratio (OR) for pCR in patients with LABC was 1.01 (95%CI (0.70-1.44)) compared with patients with IBC (p=0.979, Wald’s test). In multivariate analysis, the molecular subtype and the type of neoadjuvant chemotherapy remained significant (Table 3).

**Table 2:** Clinical outcome

Characteristics	All N=450	LABC N=306	IBC N=144	*P*-value*
Pathological complete response (pCR)				1
no	309	210	99	
yes	141	96	45	
pCR, rate	31% (95CI 27-36)	31% (95CI 26-37)	31% (95CI 24-40)	
median follow-up, months (range)	52.1 (5.49-187.37)	51.01 (5.49-187.37)	57.07 (7-172.16)	0.109
MFS event	140 (31%)	76 (25%)	64 (45%)	4.52E-05
5-year MFS	68% [95CI 63–73]	74% [95CI 68-79]	57% [95CI 49-67]	1.33E-04
LRRFS event	33 (7%)	15 (5%)	18 (13%)	6.05E-03
5-year LRRFS	92% [95CI 89-95]	95% [95CI 92-97]	86% [95CI 80-93]	3.73E-03
OS event	81 (18%)	32 (11%)	49 (34%)	5.62E-09
5-year OS	82% [95CI 78-86]	88% [95CI 83-92]	69% [95CI 62-78]	5.21E-07

* Fisher’s exact test for the discrete variables and log-rank test for survival rates.

**Table 3:** Uni-and multivariate analyses for pCR

pCR	Univariate			Multivariate		
	N	Odds ratio [CI95]	*P-*value*	N	Odds ratio [CI95]	*P-*value*
Age at diagnosis (years)	450	0.99 [0.98-1.01]	0.294			
Clinical axillary lymph node status, cN1-3 vs. cN0	445	0.88 [0.59-1.32]	0.593			
Pathological type, lobular vs. ductal	448	0.22 [0.08-0.50]	5.39E-03	448	0.44 [0.16-1.04]	0.147
Pathological type, other vs. ductal	448	0.72 [0.37-1.34]	0.401	448	0.66 [0.33-1.26]	0.301
Molecular subtype, HER2 + vs. HR+	450	4.92 [3.26-7.53]	3.84E-10	448	2.82 [1.17-6.58]	4.68E-02
Molecular subtype, TN vs. HR+	450	3.96 [2.50-6.31]	9.71E-07	448	3.55 [2.21-5.74]	1.19E-05
Pathological grade, 2 vs. 1	431	0.73 [0.37-1.54]	0.474			
Pathological grade, 3 vs. 1	431	1.58 [0.82-3.23]	0.268			
Neoadjuvant/adjuvant trastuzumab, yes vs. no	450	3.42 [2.36-4.97]	5.36E-08	448	1.76 [0.73-4.38]	0.293
Neoadjuvant chemotherapy, anthracycline & taxane vs. anthracycline	450	2.42 [1.53-3.98]	2.31E-03	448	2.18 [1.22-4.04]	3.18E-02
Stage, LABC vs. IBC	450	1.01 [0.70-1.44]	0.979	448	0.77 [0.50-1.17]	0.306

* Logistic regression analysis using the glm function in R’s statistical package.

Significance was estimated by Wald’s test specifying a binomial family for model with a logit link.

Similar comparative analyses were repeated *per* molecular subtype. In the HR+ subtype, the pCR rate was 17% in patients with IBC *versus* 14% in patients with LABC (OR=0.80, 95%CI (0.41-1.60); p=0.585, Wald’s test). In the HER2+ subtype, the respective pCR rates were 43% *versus* 49% (OR=1.25, 95%CI (0.69-2.27); p=0.538, Wald’s test). In the TN subtype, they were 40% *versus* 43% respectively (OR=1.12, 95%CI (0.56-2.30); p=0.784, Wald’s test). Thus, like in the whole population, the pCR rate was not different between IBC and LABC in each molecular subtype.

In order to further compare IBC and LABC in term of pCR, we did univariate analyses for pCR in each of them separately. The results were similar for all tested variables, except the pathological type (Supplementary Table 1). The age, cN status, and pathological grade were not associated with pCR in both IBC and LABC, whereas the molecular subtypes, delivery of neoadjuvant trastuzumab, and delivery of anthracycline and taxane-based regimen of neoadjuvant chemotherapy were associated with pCR in both IBC and LABC. By contrast, the pathological type was associated with pCR in LABC (with more pCR in ductal than lobular cancers, OR=0.16 95%CI (0.04-0.47), p=1.52E-02, Wald’s test), but not in IBC (OR=0.35, 95%CI (0.08-1.14), p=1.91E-01, Wald’s test).

### Metastasis-free survival

**Figure 1:**
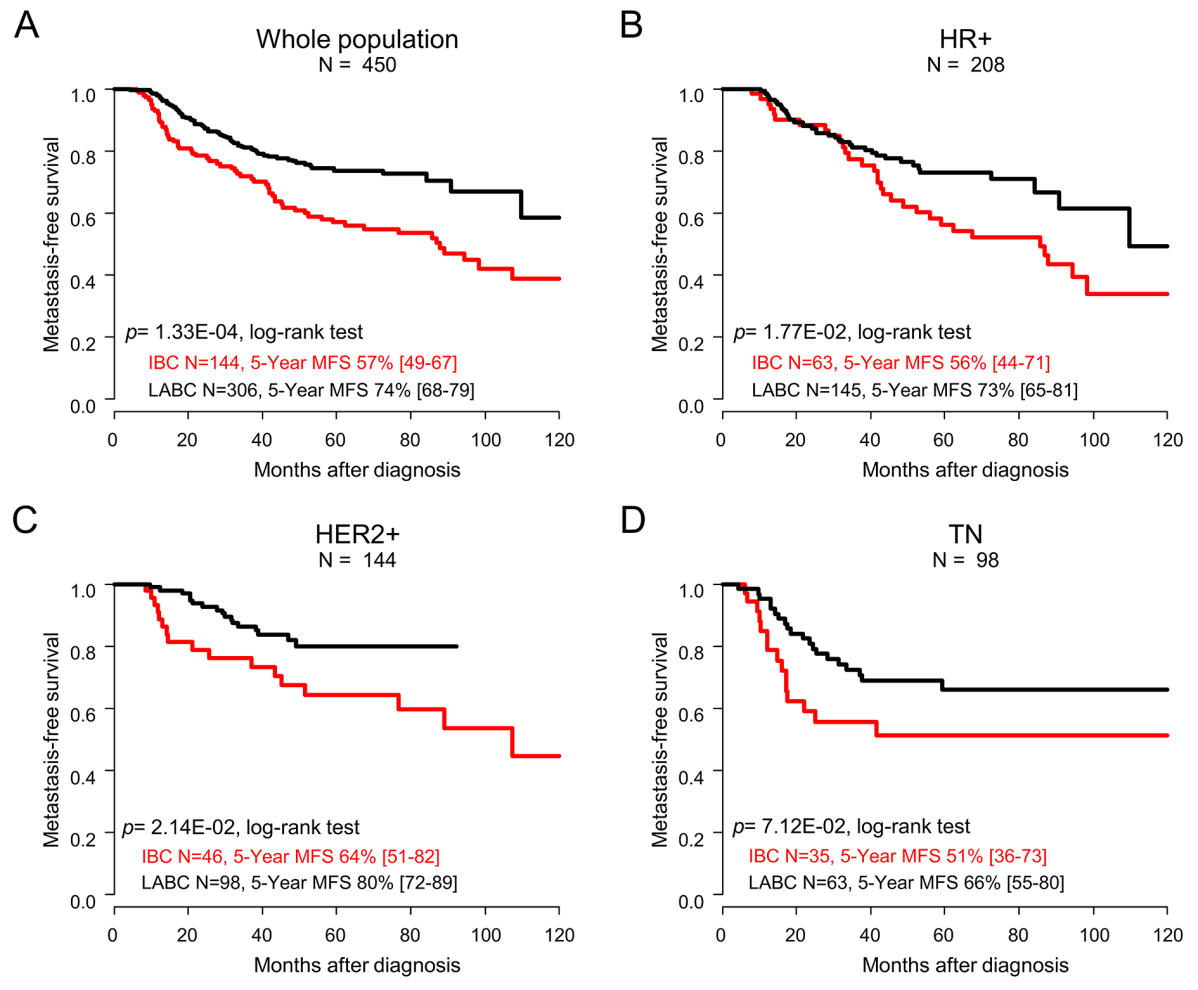
Metastasis-free survival Kaplan-Meier MFS curves comparing the IBC (red curve) *versus* LABC (black curve) patients in the whole population **(A)**, and in the different molecular subtypes: HR+ **(B)**, HER2+ **(C)**, and TN **(D)**. P-values for the log-rank test and estimations with their 95% bilateral confidence intervals are indicated.

With a median follow-up of 52.1 months (range, 5.5 to 187.4), 140 out of 450 patients (31%) experienced metastatic relapse, including 64 patients with IBC (45%) *versus* 76 patients with LABC (25%; p=4.52E-05; Fisher’s exact test). The 5-year metastasis-free survival (MFS), calculated from the date of diagnosis until the date of first distant metastasis, was 68% (95%CI (63–73)) in the whole population, and 57% (95%CI (49–67)) in patients with IBC *versus* 74% (95%CI (68-79)) in patients with LABC (p=1.33E-04; log-rank test; Figure 1A, Table 2). In univariate prognostic analyses, the IBC/LABC variable was significant with a Hazard Ratio (HR) for relapse equal to 0.35 (95%CI (0.21-0.61); p=1.56E-04; Wald’s test, Table 4) in the LABC group compared with the IBC group. In addition, the age, pathological type and grade, delivery of neoadjuvant/adjuvant trastuzumab, of anthracycline-taxane-based neoadjuvant chemotherapy, of adjuvant radiotherapy and hormone therapy, and achievement of pCR were associated with MFS (Table 4). In multivariate analysis, the IBC/LABC variable remained significant, as did the achievement of pCR. Analysis *per* molecular subtype confirmed the worse prognosis of patients with IBC compared with patients with LABC in each subtype (Figure 1B-1D). Of note, the patients with TN IBC displayed shorter MFS than patients with HR+ IBC or HER2+ IBC, as observed in LABC.

**Table 4:** Univariate analysis for MFS, LRRFS and OS

MFS	Univariate			Multivariate		
	N	Hazard ratio [CI95]	*P*-value	N	Hazard ratio [CI95]	*P*-value
Age at diagnosis (years)	450	1.03 [1.01-1.04]	6.77E-04	414	1.02 [1.01-1.04]	4.14E-03
Clinical axillary lymph node status, cN1-3 vs. cN0	445	1.32 [0.87-2.02]	0.193			
Pathological type, lobular vs. ductal	448	1.35 [0.80-2.27]	2.20E-02	414	1.49 [0.84-2.63]	0.173
Pathological type, other vs. ductal		0.29 [0.11-0.78]		414	0.19 [0.05-0.76]	1.93E-02
Molecular subtype, HER2 + vs. HR+	450	0.75 [0.50-1.14]	0.103			
Molecular subtype, TN vs. HR+		1.26 [0.84-1.89]				
Pathological grade, 2 vs. 1	431	1.35 [0.60-3.02]	9.02E-03	414	1.39 [0.60-3.24]	0.441
Pathological grade, 3 vs. 1		2.21 [1.02-4.79]		414	2.33 [1.01-5.38]	4.67E-02
Adjuvant radiotherapy, yes vs. no	444	0.31 [0.14-0.71]	5.60E-03	414	0.34 [0.14-0.80]	1.35E-02
Adjuvant hormone therapy, yes vs. no	434	0.57 [0.41-0.80]	1.04E-03	414	0.50 [0.34-0.72]	1.98E-04
Neoadjuvant/adjuvant trastuzumab, yes vs. no	450	0.36 [0.21-0.61]	1.88E-04	414	0.50 [0.28-0.91]	2.29E-02
Neo-adjuvant chemotherapy, anthracycline & taxane vs. anthracycline	450	0.43 [0.30-0.61]	2.66E-06	414	0.72 [0.47-1.09]	0.122
Stage, LABC vs. IBC	448	0.35 [0.21-0.61]	1.56E-04	414	0.67 [0.46-0.99]	4.29E-02
Pathological complete response, yes vs. no	450	0.52 [0.37-0.73]	1.71E-04	414	0.47 [0.29-0.76]	2.24E-03

LRRFS	Univariate			Multivariate		
	N	Hazard ratio [CI95]	*P*-value	N	Hazard ratio [CI95]	*P*-value
Age at diagnosis (years)	450	1.01 [0.98-1.04]	0.347			
Clinical axillary lymph node status, cN1-3 vs. cN0	445	1.78 [0.69-4.62]	0.235			
Pathological Stage, lobular vs. ductal	448	0.30 [0.04-2.20]	0.246			
Pathological Stage, other vs. ductal		0.29 [0.04-2.12]				
Molecular subStage, HER2 + vs. HR+	450	1.16 [0.51-2.64]	0.311			
Molecular subStage, TN vs. HR+		1.87 [0.82-4.27]				
Pathological grade, 2 vs. 1	431	0.59 [0.11-3.04]	1.96E-02	414	0.43 [0.08-2.35]	0.329
Pathological grade, 3 vs. 1		2.20 [0.52-9.32]		414	1.65 [0.38-7.12]	0.502
Adjuvant radiotherapy, yes vs. no	444	0.14 [0.04-0.45]	1.09E-03	414	0.08 [0.02-0.31]	1.78E-04
Adjuvant hormone therapy, yes vs. no	434	0.48 [0.24-0.98]	4.25E-02	414	0.58 [0.27-1.22]	0.149
Neoadjuvant/adjuvant trastuzumab, yes vs. no	450	0.82 [0.35-1.9]	0.644			
Neoadjuvant chemotherapy, anthracycline & taxane vs. anthracycline	450	0.34 [0.17-0.69]	2.56E-03	414	0.41 [0.18-0.93]	3.33E-02
Stage, LABC vs. IBC	450	0.37 [0.19-0.75]	5.24E-03	414	0.49 [0.22-1.11]	0.088
Pathological complete response, yes vs. no	450	0.49 [0.2-1.18]	0.112			

OS	Univariate			Multivariate		
	N	Hazard ratio [CI95]	*P*-value	N	Hazard ratio [CI95]	*P*-value
Age at diagnosis (years)	450	1.02 [1.00-1.03]	0.122			
Clinical axillary lymph node status, cN1-3 vs. cN0	445	1.68 [0.92-3.04]	0.090			
Pathological Stage, lobular vs. ductal	448	1.24 [0.62-2.50]	0.370			
Pathological Stage, other vs. ductal		0.54 [0.20-1.47]				
Molecular subStage, HER2 + vs. HR+	450	0.75 [0.43-1.32]	2.55E-02	414	0.78 [0.37-1.61]	0.496
Molecular subStage, TN vs. HR+		1.67 [1.01-2.77]		414	0.85 [0.43-1.68]	0.643
Pathological grade, 2 vs. 1	431	1.69 [0.50-5.69]	9.63E-03	414	1.66 [0.49-5.65]	0.420
Pathological grade, 3 vs. 1		3.27 [1.02-10.5]		414	2.64 [0.81-8.57]	0.106
Adjuvant radiotherapy, yes vs. no	444	0.15 [0.06-0.35]	1.07E-05	414	0.16 [0.07-0.38]	4.41E-05
Adjuvant hormone therapy, yes vs. no	434	0.42 [0.27-0.66]	1.41E-04	414	0.38 [0.21-0.69]	1.58E-03
Neoadjuvant/adjuvant trastuzumab, yes vs. no	450	0.22 [0.09-0.55]	1.13E-03	414	0.39 [0.12-1.23]	0.108
Neoadjuvant chemotherapy, anthracycline & taxane vs. anthracycline	450	0.35 [0.22-0.54]	4.18E-06	414	0.64 [0.38-1.07]	0.089
Stage, LABC vs. IBC	450	0.33 [0.21-0.52]	1.62E-06	414	0.47 [0.28-0.77]	2.51E-03
Pathological complete response, yes vs. no	450	0.57 [0.33-0.98]	4.17E-02	414	0.63 [0.35-1.13]	0.122

### Loco-regional relapse-free survival

**Figure 2:**
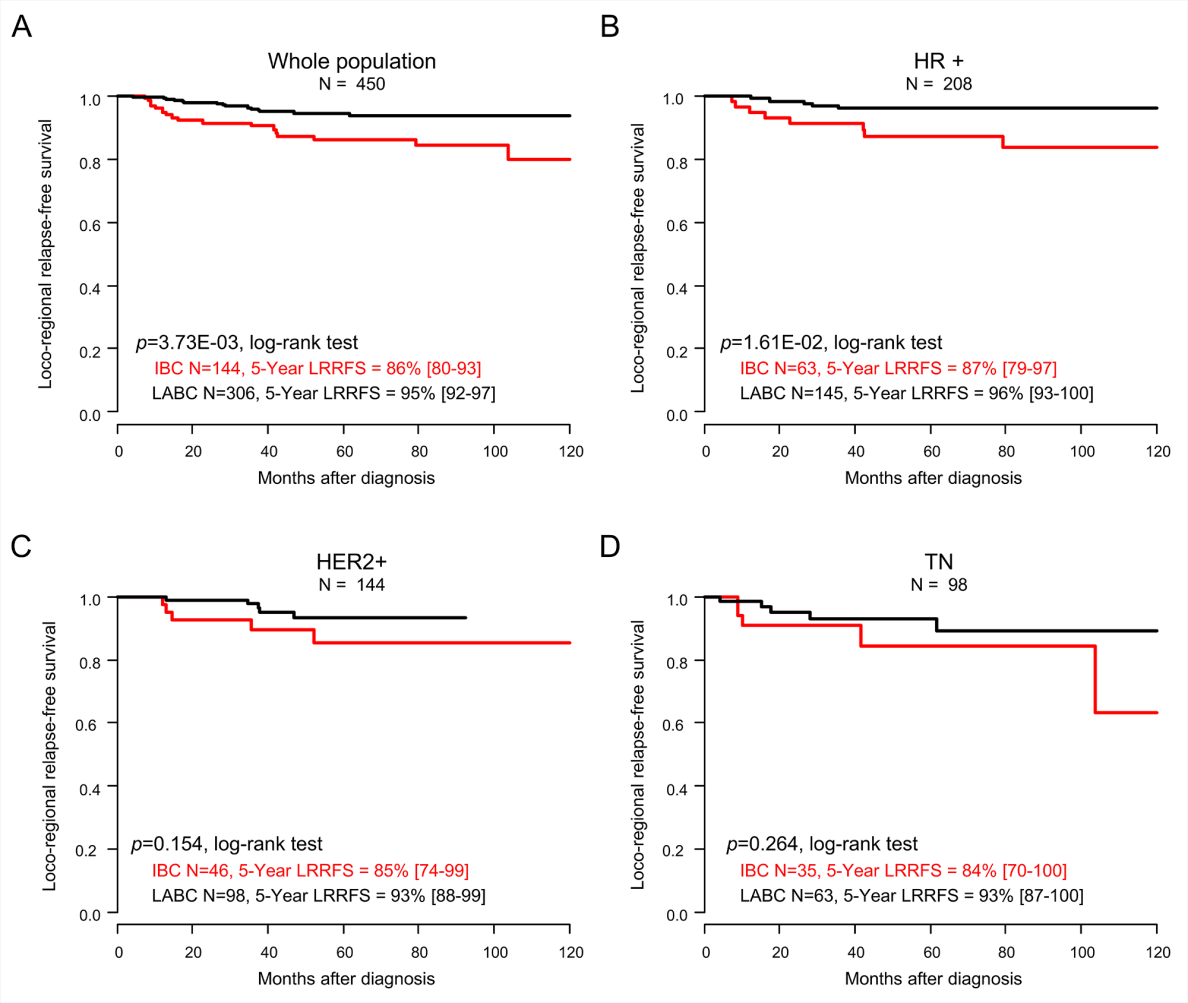
Loco-regional relapse-free survival Kaplan-Meier LRRFS curves comparing the IBC (red curve) *versus* LABC (black curve) patients in the whole population **(A)**, and in the different molecular subtypes: HR+ **(B)**, HER2+ **(C)**, and TN **(D)**. P-values for the log-rank test and estimations with their 95% bilateral confidence intervals are indicated.

Similar results were observed regarding loco-regional relapse-free survival (LRRFS). Thirty-three patients experienced loco-regional relapse (7%), including 18 with IBC (13%) and 15 with LABC (5%; p=6.05E-03; Fisher’s exact test). The 5-year LRRFS, calculated from the date of diagnosis until the date of first local and/or regional relapse, was 92% (95%CI (89-95)) in the whole population, and 86% (95%CI (80-93)) in patients with IBC *versus* 95% (95%CI (92-97)) in patients with LABC (p=3.73E-03; log-rank test; Figure 2A, Table 2). In univariate prognostic analyses, the IBC/LABC variable was associated with LRRFS, as well as the pathological grade, delivery of neoadjuvant/adjuvant trastuzumab, anthracycline-taxane-based neoadjuvant chemotherapy, adjuvant radiotherapy and hormone therapy (Table 4). The Hazard Ratio (HR) for loco-regional relapse was equal to 0.37 (95%CI (0.19-0.75); p=5.24E-03; Wald’s test) in the LABC group compared with the IBC group (Table 4). In multivariate analysis, the IBC/LABC variable tended to be significant (p=0.088). Analysis *per* molecular subtype showed worse 5-year LRRFS in patients with IBC compared with patients with LABC in each subtype, although the difference was not significant, likely because of the relative small number of events (Figure 2B-2D).

### Overall survival

**Figure 3:**
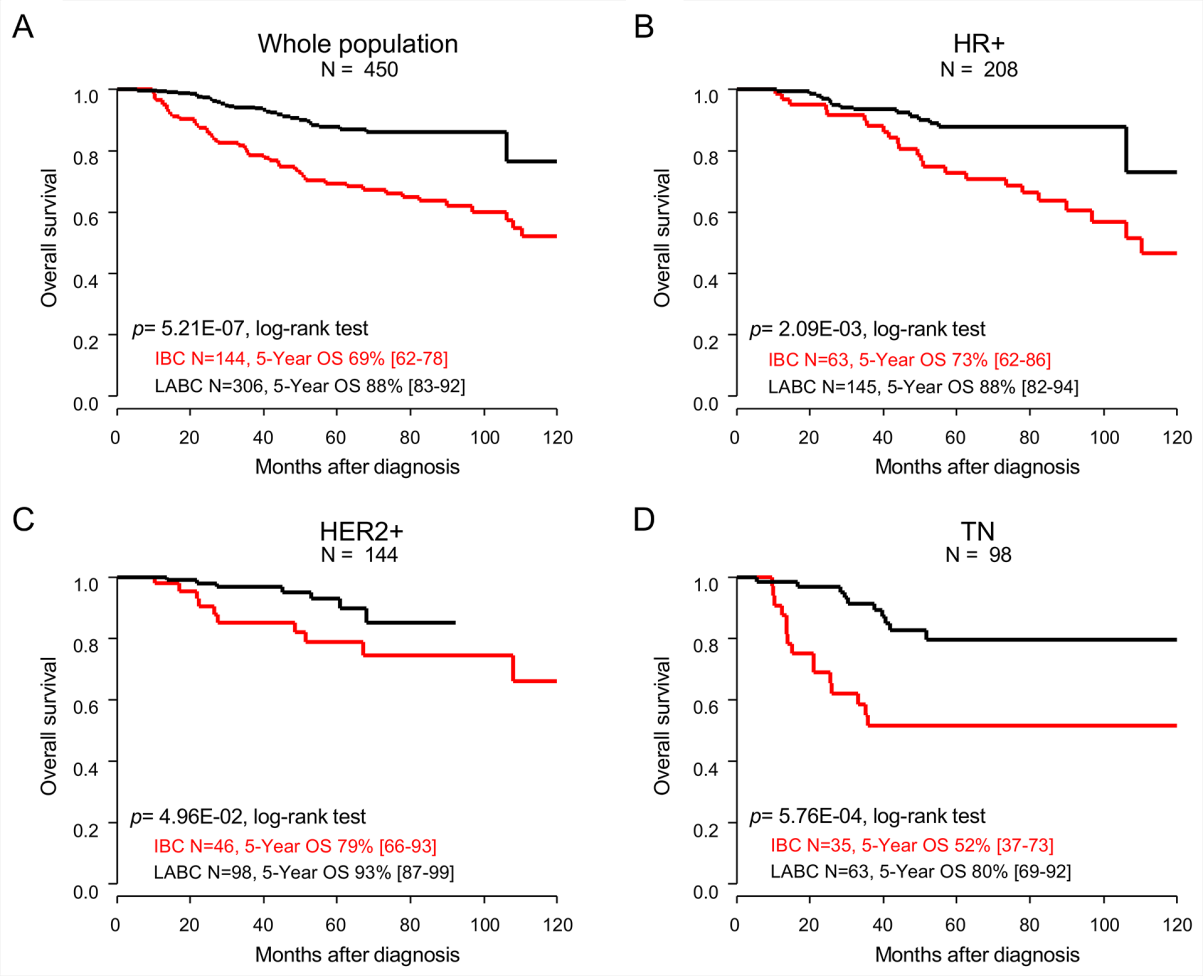
Overall survival Kaplan-Meier OS curves comparing the IBC (red curve) *versus* LABC (black curve) patients in the whole population **(A)**, and in the different molecular subtypes: HR+ **(B)**, HER2+ **(C)**, and TN **(D)**. P-values for the log-rank test and estimations with their 95% bilateral confidence intervals are indicated.

Similar results were also observed regarding OS. Eighty-one patients died (18%), including 49 with IBC (34%) and 32 with LABC (10.5%; p=5.62E-09; Fisher’s exact test). The 5-year overall survival (OS), from the date of diagnosis until the date of death whatever its cause, was 82% (95%CI (78-86)) in the whole population, and 69% (95%CI (62-78)) in patients with IBC *versus* 88% (95%CI (83-92)) in patients with LABC (p=5.21E-07; log-rank test; Figure 3A, Table 2). In univariate prognostic analyses, the IBC/LABC variable was associated with OS, as well as the molecular subtypes, pathological grade, delivery of neoadjuvant/adjuvant trastuzumab, of anthracycline-taxane-based neoadjuvant chemotherapy, of adjuvant radiotherapy and hormone therapy, and achievement of pCR (Table 4). The Hazard Ratio (HR) for death was equal to 0.33 (95%CI (0.21-0.52); p=1.62E-06; Wald’s test) in the LABC group compared with the IBC group (Table 4). In multivariate analysis, the IBC/LABC variable remained significant. Here too, the analyses *per* molecular subtype confirmed the worse prognosis of patients with IBC compared with patients with LABC in each subtype (Figure 3B-3D). Here too, the patients with TN IBC displayed shorter OS than patients with HR+ IBC or HER2+ IBC, as observed in LABC patients.

## DISCUSSION

IBC is the most aggressive form of breast cancer. Compared to LABC, survival is shorter, whereas the treatment is similarly based on neoadjuvant anthracycline-based chemotherapy followed by surgery. Better understanding the causes of its poor prognosis is crucial. Today, it remains unclear whether IBC is less chemosensitive than LABC. In this retrospective study of 450 patients treated in our institution, including 144 with IBC and 306 with LABC, we showed that the pCR rate after anthracycline-based neoadjuvant chemotherapy was similar between IBC and LABC, although the MFS, LRRFS, and OS were different. To our knowledge, this is the largest unicentric series comparing both the pCR rate to neoadjuvant chemotherapy and the survival rates between patients with IBC and with LABC.

The retrospective nature of our study was justified by the scarcity of prospective data in this field, due to at least two reasons: the scarcity of IBC and dedicated clinical trials, and its differences with non-IBC leading in most of the cases to its exclusion from breast cancer clinical trials. IBC and LABC patients were defined according to clinical criteria in the AJCC/TNM staging. For IBC, the definition is consensual [1]. For LABC, the definition is less consensual in the literature and differs according to the clinical trials; here, we used the definition used in the SWOG clinical trials of neoadjuvant chemotherapy [13, 14]. Of note, the comparative analysis of pCR and survivals in IBC (N=144) and LABC (N=123) defined according to the definition used in the NOAH clinical trial [15] showed results similar to our current results, with similar pCR rates but different survivals (data not shown). Our primary endpoint, pathological response to neoadjuvant chemotherapy, is an intermediate endpoint allowing a fast assessment of the chemosensitivity more accurate than the clinical response [16]. The pCR definition was based on eradication of invasive tumor from both breast and lymph nodes, which is a better surrogate endpoint for survival than eradication in the breast alone [10]. The large size of our series allowed multivariate analyses and the comparison of pCR and survivals *per* molecular subtype.

Our two groups (IBC and LABC) were balanced in terms of patients’ age, menopausal status, pathological type, and molecular subtypes. As expected, there were more tumors with emboli in dermal lymphatic vessels in the IBC group, since emboli represent the pathological hallmark of disease [17]. The number of grade 2-3 tumors was similar in IBC and LABC. Regarding the neoadjuvant treatment, there was an unbalance in favor of the LABC group in terms of neoadjuvant trastuzumab and of anthracycline and taxane-based regimen, which have been shown to increase the pCR rate when compared to absences of trastuzumab [15] and of taxane [18, 19]. As expected, mastectomy was much more frequent in the IBC group, and there was no difference in term of adjuvant radiotherapy and hormone therapy. The pCR rate was 31% in the whole series, and perfectly similar between IBC and LABC (31%), despite the unbalance in detriment of the IBC group in terms of neoadjuvant trastuzumab and anthracycline-taxane-based combination, suggesting at least that IBC is not less chemosensitive than LABC. Such rates are close to those previously reported in IBC in the French Pegase trials, with non-centrally reviewed pCR rates of 32% and 20.1% in the anthracycline-based Pegase 02 [20] and 07 [21] trials respectively, and 35% in the anthracycline/taxane-based Pegase 05 trial [22]. They are in agreement with the rates, ranging from 17 to 39%, reported in a literature review [1], but slightly superior to those reported in the largest unicentric series recently reported by MDA Anderson. In this retrospective series including 527 patients treated between 1989 and 2011, the pCR rate was 15.2% in the whole population, 7.5% in HR+ patients, 24.7% in HER2+ patients, and 12.4% in TN patients. In our present series, despite similar pCR rates in IBC and LABC and the prognostic value of pCR, patients with IBC displayed shorter 5-year MFS (57%), LRRFS (86%), and OS (69%) than patients with non-IBC (74%, 95%, and 88%, respectively). Such survival rates were in agreement with literature for both IBC and non-IBC [23-25], and confirmed the poorer prognosis of IBC. This poor prognosis is confirmed in retrospective series with a long follow-up. For example, in the unicentric NCI series [26], the 15-year OS was 20% for IBC patients after a median follow-up of 16.8 years. In a more recent large multicentric study including 673 IBC patients, the 10-year OS was 41% in stage III patients [27]. Whereas the molecular subtype was associated with OS in our univariate analysis (a trend was observed regarding MFS) with shorter survival in patients with TN breast cancer, it lost its prognostic value in multivariate analysis. By contrast and as expected, the IBC phenotype remained an independent prognostic feature in multivariate analysis, and its prognostic value was observed in each molecular subtype, in which the pCR rate was however similar between IBC and non-IBC.

During the last ten years, several prospective, large-scale, randomized trials of neoadjuvant chemotherapy in LABC have been reported and included some patients with IBC. However, in most of them [28-33], the detailed results in term of pCR for IBC and LABC separately were not provided, preventing any comparison. For example, the NOAH trial [15] showed that neoadjuvant/adjuvant trastuzumab improved event-free survival in 235 patients with HER2-positive LABC (N=172) or IBC (N=63), and doubled the pCR rate after neoadjuvant chemotherapy (from 19 to 38%); but the pCR rate in LABC and IBC separately was not provided. Similarly, in the NeoSphere trial [34], that included 29 patients with IBC out of the 417 enrolled patients, the pCR rate was not detailed for IBC, LABC and OBC. By contrast, in a few trials, the information was available, and although the comparison LABC/IBC was not planned in general, it provided interesting data. In the GeparTrio trial [35], patients were randomly assigned, after stratification by stage, to six or eight cycles of docetaxel/doxorubicin/cyclophosphamide (TAC) or to two cycles of TAC followed by four cycles of vinorelbine/capecitabine: 93 patients with IBC and 194 patients with LABC were treated with the same regimen as 1,777 patients with OBC. The comparison of pCR between IBC and LABC treated as a subgroup and OBC was pre-planned. The pCR rate (ypT0/Tis-ypN0) was not different between IBC (17.2%) and LABC (13.8%; p=0.54). In the SWOG 0012 trial [13], patients with IBC (N=116) or LABC (N=256) were randomly assigned to 21-day doxorubicin-cyclophosphamide (AC) regimen administered for five cycles (standard arm) *versus* weekly doxorubicin and daily oral cyclophosphamide administered with granulocyte colony-stimulating factor support for 15 weeks (continuous arm). All patients had subsequent weekly paclitaxel for 12 weeks before surgery. The pCR rate (ypT0/Tis-ypN0) was not different between IBC (19.8%) and LABC (23.7%; p=0.49), although the 5-year DFS and OS were longer in patients with LABC. In the SWOG S0800 trial [14], patients with LABC (N=187) or IBC (N=24) were randomly allocated to three neoadjuvant chemotherapy arms: nab-paclitaxel with concurrent bevacizumab followed by AC, or nab-paclitaxel followed by AC, or AC followed by nab-paclitaxel. The pCR rate (ypT0/Tis-ypN0) was not different between IBC (21%) and LABC (29%; p=0.48). In the GeparQuatro trial [36] that tested the addition of capecitabine to or the prolongation of duration of neoadjuvant epirubicin-cyclophosphamide plus docetaxel in large operable or locally advanced breast cancers, the pCR rate was not different between IBC (N=110, pCR=19%) and cT4a-c LABC (N=118, pCR=14% ; p=0.28). Thus, only one (GeparTrio) of those prospective trials provided direct and pre-planned comparison of pathological response between IBC and non-IBC. Here, our retrospective study confirms similar response profiles between both stages, but in a larger and unicentric series of IBC patients.

Altogether, these results, including ours, suggest that the poorer survival of IBC patients, compared to LABC patients, is more due to the higher metastatic risk of disease and/or other feature(s) than to a lesser chemosensitivity. This peculiar metastatic potential of IBC was already demonstrated several years ago by the very low 5-year survival rates (<5%) observed when the patients were treated with surgery and/or radiotherapy only [37]. It is also suggested by the fact that several prognostic factors of non-IBC do not work well in IBC [38, 39], suggesting that different mechanisms are involved in the metastatic process. Finding similar pCR rates in IBC and non-IBC may appear surprising given the classical biological differences between both phenotypes [40]. Indeed, IBC is classically more frequently associated with molecular alterations related to chemosensitivity [41]: high proliferation, high grade, HR-negativity, HER2-positivity, TN and HER2+ molecular subtypes, and *TP53* mutations. However, in our present series, the IBC and non-IBC groups were well balanced with respect to these features, and the pCR rates were identical in IBC and LABC not only in the whole cohort, but also in each molecular subtype. Similarly, the secondary analysis of the GeparTrio trial [35] showed that even if the IBC and LABC groups combined (12.2%) had a lower pCR rate than patients with OBC (23.6%), the difference was not significant in multivariate analysis, suggesting that tumor stage itself was not an independent predictor of pCR and that the different pCR rates observed were likely due to heterogeneity in tumor features such as grade or hormone receptor status. We also compared the factors predictive for pCR in IBC and non-IBC and showed that all tested variables, except the pathological type, behaved in the same way, further suggesting that the achievement of a response to chemotherapy involves the same mechanisms in both phenotypes. This is consistent with our previous genomics study [38], in which we had shown that a gene expression signature associated with pCR in IBC worked well in non-IBC and reciprocally, that signatures associated with pCR in non-IBC worked well in IBC. The only tested predictive variable that behaved differently in our present univariate analyses was the pathological type: in non-IBC, it was associated with pCR with, as expected [42], more pCR in ductal than lobular cancers, whereas it was not in IBC. Of course, the small number of lobular IBC samples precludes any definitive conclusion, but this observation reminds a recent study from the MDA Anderson Cancer Center [39], which showed that the lobular histology did not have any prognostic value in IBC patients, unlike in patients with non-IBC.

In conclusion, our data show that anthracycline-based neoadjuvant chemotherapy provides similar patterns of pathological response in IBC and in LABC, suggesting that IBC is not less sensitive to chemotherapy than LABC. However, the long-term survival is shorter in patients with IBC. The strength of our study lies in its originality and the size of our IBC series. To our knowledge, it is the largest unicentric in term of IBC (more than 140 cases) that compares both the pCR and survival rates in patients with IBC and with LABC. Its main limitation lies in its retrospective nature and associated biases. However, our population was more likely representative of a “real-life” cohort, with some patients not receiving the complete treatment or ideal care because of their own choice, advanced age, or comorbidities. Furthermore, no direct comparison planned in a prospective trial combining pCR and survival data and concerning more than one hundred IBC patients has been published to date and it is very likely that there will never be such randomized prospective trial in the future. In this context, a meta-analysis of prospective clinical trials of neoadjuvant chemotherapy including both IBC and non-IBC is warranted for helping to answer this issue.

## MATERIALS AND METHODS

### Study design and inclusion criteria

We retrospectively selected the patients with a diagnosis of breast cancer included in our institutional database. The study was approved by our Institutional Review Board. The main inclusion criteria included the pathological diagnosis of invasive primary carcinoma of the breast based on tumor biopsy, non-metastatic at time of diagnosis (considering the ipsilateral supraclavicular lymph node(s) as metastasis(ses)), treated with neoadjuvant anthracycline-based chemotherapy followed by surgery (lumpectomy or mastectomy and axillary lymph node dissection), and documentation of pathological response. The variables collected and analyzed included the patients’ age at diagnosis, the menopausal status, the cT and cN classes, the pathological type and grade, the ER and PR immunohistochemistry (IHC) status (with a 10% of stained tumor cells as threshold of positivity), the HER2 status (determined according to French guidelines by IHC +/- fluorescent *in situ* hybridization), the existence of neoplastic emboli in dermal lymphatic vessels, the treatment received (neoadjuvant chemotherapy regimen, definitive surgery, adjuvant radiotherapy and hormone therapy, and neoadjuvant/adjuvant trastuzumab), and the clinical outcome in terms of pCR and survival. Identification of molecular subtypes is most precise using gene expression profiling [43]. But such assay was not available for most of patients. The molecular subtype of tumors was thus defined as follows: HR+ for ER+ and/or PR+ and HER2- tumors, HER2+ for HER2+ tumors, and triple negative (TN) for ER-, PR- and HER2- tumors. Ki67 IHC status was unavailable for most of samples, thus preventing us to define the luminal A and luminal B subtypes [44]. When necessary, missing data were completed by review of patients’ medical files.

The patients were divided in two groups (IBC and LABC) based on the clinical criteria of the four successive AJCC/TNM cancer staging editions (4^th^ to 7^th^ editions) concerned by the two-decade period covered by our series. These four editions have changed over this period regarding the N2 and N3 classes and the M1 class. Thus, to avoid any bias related to the use of different staging editions, we excluded from analyses the patients with ipsilateral supraclavicular lymph node(s) at diagnosis, and we pooled the N2 and N3 tumors in the cN1-3 class in statistical analyses. IBC were defined by inflammatory clinical signs involving more than one-third of the breast (cT4d classification). LABC were defined as non-IBC with a tumor diameter superior to 5 cm (cT3), or a tumor involving the skin or muscle (cT4a-c), or a tumor diameter between 2 and 5 cm (cT2) associated with metastases to ipsilateral regional lymph nodes (axillary, internal mammary, infraclavicular, or supraclavicular: cN1-3).

### Clinical outcome

The primary endpoint was the proportion of patients who achieved a pathological complete response (pCR) after neoadjuvant chemotherapy. Post-chemotherapy mastectomy or lumpectomy and axillary lymph node dissections were examined by pathologists to describe the pathological response. pCR was defined as absence of invasive tumor in breast and axillary lymph nodes (ypT0/Tis-ypN0). The secondary endpoints included the metastasis-free survival (MFS), calculated from the date of diagnosis until the date of first distant metastasis, the loco-regional relapse-free survival (LRRFS), calculated from the date of diagnosis until the date of first local and/or regional relapse, and overall survival (OS), from the date of diagnosis until the date of death whatever its cause. The follow-up was measured from the date of diagnosis to the date of last news by using the reverse Kaplan-Meier method. Data concerning patients without death or metastatic relapse at last follow-up were censored.

### Statistical analysis

Patients’ characteristics were compared with the Fisher’s exact test and the Mann-Whitney test. Survivals were estimated using the Kaplan-Meier method and curves were compared with the log-rank test. Univariate and multivariate pCR analyses were done using a logistic regression analysis. Univariate and multivariate survival analyses were done using Cox regression analysis. Variables tested in univariate analyses included age at diagnosis, clinical axillary lymph node status, pathological type and grade, ER, PR and HER2 status, molecular subtype, neoadjuvant chemotherapy regimen, neoadjuvant/adjuvant trastuzumab. Additional variables for univariate survival analyses included adjuvant radiotherapy and hormone therapy, and pathological response. Multivariate analyses were conducted by including variables significant in univariate analysis and the IBC/LABC type and were limited to patients for which all variables tested were specified. All statistical tests were two-sided at the 5% level of significance. Analyses were done in the R software and associated packages (version 2.15.2).

## SUPPLEMENTARY MATERIALS TABLE





## Abbreviations

AJCC TNM: American Joint Committee on Cancer Tumor Node Metastasis
CI: confidence interval
DFS: disease-free survival
ER: estrogen receptor
HER-2: human epidermal growth factor receptor-2
HR: hormone receptor
IBC: inflammatory breast cancer
IHC: immunohistochemistry
LABC: locally advanced breast cancer
MFS: metastasis free survival
Non-IBC: non-inflammatory breast cancer
OBC: operable breast cancer
OR: odds ratio
OS: overall survival
pCR: pathological complete response
PR: progesterone receptor
TN: triple negative
