# Supplementary material for: Similar response profile to neoadjuvant chemotherapy, but different survival, in inflammatory versus locally advanced breast cancers
Source: Oncotarget. 2017 Jul 31;8(39):66019–32. doi: 10.18632/oncotarget.19732 (PMC5630389; doi:10.18632/oncotarget.19732)
Supplement: Supplementary file 1 [file oncotarget-08-66019-s001.pdf]

Similar response profile to neoadjuvant chemotherapy, but different survival, in inflammatory *versus* locally advanced breast cancers

SUPPLEMENTARY MATERIALS

Supplementary Table 1: Univariate analyses for pCR in IBC and in non-IBC

See Supplementary File 1
